# *Beilstein Journal of Organic Chemistry*: Open Access comes of age in Organic Chemistry

**DOI:** 10.3762/bjoc.8.145

**Published:** 2012-08-13

**Authors:** Peter H Seeberger

**Affiliations:** 1 Max-Planck-Institute of Colloids and Interfaces, Department of Biomolecular Systems, Am Mühlenberg 1, 14476 Potsdam, Germany

The *Beilstein Journal of Organic Chemistry* (BJOC) was started in 2005 [1] as the first Open Access journal of the Beilstein-Institut. The Beilstein-Institut is a non-profit foundation that carries out projects supporting advances in chemical sciences and related areas, particularly through information and communication. The aim of the *Beilstein Journal of Organic Chemistry* has been to provide a new and lasting service to enable the scientific community to publish and read high-quality scientific articles, without being hindered by a subscription pay-wall, or by budget limitations.

The Beilstein-Institut funds the Journal completely, so there are no subscriptions, or author fees, no page or color charges. The scientific community plays a significant role in the editing of the Journal; the large editorial board, currently 29 scientists, means that the submitted manuscripts are handled by experts in the field and it allows the *Beilstein Journal of Organic Chemistry* to be represented in many countries. The editors also play a significant role in communicating the advantages of the Journal to colleagues, and in the selection of new Thematic Series, which have been particularly successful for the Journal. All articles are permanently stored in public archives, such as PubMed Central, and indexed by scientific databases.

## Impact factor

From the very beginning of the Journal, it was clear just how important impact factors have become. It is not just an interesting metric for librarians to compare journals and articles, but it has become more widely used by funding agencies, universities and the like as a measure of academic prowess and is therefore used in decision making metrics for grant applications, tenure and promotions. The merits of this practice will not be addressed here. It was the aim of the *Beilstein Journal of Organic Chemistry* from the beginning to have a good impact factor, and we are now delighted that it has steadily increased over the years to reach 2.517 in 2011 [2].

## Access counts

Other metrics for assessing the impact of journals or articles are the access counts and/or number of downloads. The *Beilstein Journal of Organic Chemistry* has seen continual growth in this area as well. We have implemented strict filtering to exclude repeated views and accesses by robots when counting the HTML full text accesses and PDF downloads (we have set the time filters for multiple accesses as recommended by the COUNTER code of practice [3]). Currently we are approaching 50,000 per month, and have over 1.7 million accesses in total. As an Open Access journal, BJOC allows easy access for internet search engines to all articles in full, and just as importantly allows users to gain ready access to the results by a simple mouse click.

## Mobile access

The latest mobile devices are able to be used for accessing web-content with similar performance to that of most desktop computers. This year, we brought out the Beilstein Journals App for Android and iOS smartphones and tablets [4–5]. Whilst on smaller devices, a two-column PDF designed for print purposes does not make easy reading, the full-text HTML display is well usable for scientific papers. We are already looking at other formats, such as ePub [6], which will allow reflowing of the text in a more functional way, and which additionally would allow storage for offline reading in eBook readers. It will not be long before the use of mobile devices to gain access to scientific papers will become as commonplace for scientists as downloading music has become for the average internet user. Our aim with the mobile devices is to re-engage the reader in such a way that emulates the best aspects of the printed issue, with the ability to flick through the pages, and combine it with the technological advantages of world-wide instant access.

## Quality assurance

Each article published in the *Beilstein Journal of Organic Chemistry* has undergone rigorous internal and external review. The Beilstein-Institut has an internal editorial office for its journals, comprising a team of highly qualified scientists. The internal editorial work involves checking for plagiarism, novelty of compounds, inconsistencies and errors, and also the copyediting and laying out of the final article. The internal editors work in close contact with the external editorial board, whose expertise in specific fields of organic chemistry ensures that the manuscripts published contain novel and contemporary relevant scientific research results.

## Combating plagiarism

Plagiarism is a problem that all publishers, universities and companies have to tackle. Since May 2011 we routinely check all incoming articles for plagiarism using CrossCheck [7] and other search systems. We find instances of “copy and paste” in a significant number of submissions. Major issues result in a rejection and possible further sanctions. For a useful discussion and information on plagiarism and how to go about dealing with it see, for example, the committee on publication ethics (COPE) [8] and Roig [9].

## Beilstein TV

A further project of the Beilstein-Institut, which accompanies the journals and has valuable synergy effects, is the scientific video project – Beilstein TV [10]. Here scientists are invited to participate in the production of video films, in which they either show experimental methods in their laboratory, hold a filmed lecture, or participate in an interview or commentary. The Beilstein-Institut uses professional video production teams to ensure that the finished product is of high quality. The scientists are responsible for the scientific content and quality themselves. Of particular note are the lab videos that are made to correspond to a paper published in the *Beilstein Journal of Organic Chemistry* or in the *Beilstein Journal of Nanotechnology*. This combination allows scientists to use a visual medium to go further in the explanation of their work than is otherwise possible on paper. This can be useful in terms of overviews and perspectives, as well as for showing hands-on, just how certain experiments work or need to be set up and carried through.

## Outlook

In 2012 we have shown that the *Beilstein Journal of Organic Chemistry* has come of age as an Open Access journal. It has been accepted by the scientific community, it is read worldwide, and is rising in its impact as judged by the citations published. The combination of the Journal with mobile access, videos and further projects bodes well for the future. The *Beilstein Journal of Organic Chemistry* is now established as the leading open access journal in its field, and is well placed to not only take advantage of future scientific and technical advances in publishing or media, but is flexible enough to lead the way amongst comparable journals. We are planning to expand our editorial board into other subject areas and countries, to provide the organic chemistry community with further Thematic Series of contempory interest and to develop innovative methods of communicating organic chemistry. We are looking forward with our authors, editors, reviewers and readers to an even more successful time ahead.

Peter H. Seeberger

Potsdam, August 2012
